# Long non-coding RNA NORAD contributes to the proliferation, invasion and EMT progression of prostate cancer via the miR-30a-5p/RAB11A/WNT/β-catenin pathway

**DOI:** 10.1186/s12935-020-01665-2

**Published:** 2020-11-27

**Authors:** Yunxia Zhang, Yang Li

**Affiliations:** 1 Department of Nursing, Huaihe Hospital of Henan University, Kaifeng, 475000 People’s Republic of China; 2 The Second Ward, Department of Urinary Surgery, Huaihe Hospital of Henan University, Kaifeng, 475000 People’s Republic of China

**Keywords:** Prostate cancer, lncRNA NORAD, miR-30a-5p, RAB11A, Wnt/β-catenin, Epithelial–mesenchymal transition, Proliferation, Apoptosis

## Abstract

**Background:**

Prostate cancer (PC) is common male cancer with high mortality worldwide. Emerging evidence demonstrated that long noncoding RNAs (lncRNAs) play critical roles in various type of cancers including PC by serving as competing endogenous RNAs (ceRNAs) to modulate microRNAs (miRNAs). LncRNA activated by DNA damage (NORAD) was found to be upregulated in PC cells, while the detailed function and regulatory mechanism of NORAD in PC progression remains largely unclear.

**Methods:**

Expression of NORAD in PC tissues and cell lines were detected by real-time quantitative PCR (qRT-PCR). NORAD was respectively overexpressed and knocked down by transfection with pcDNA-NORAD and NORAD siRNA into PC-3 and LNCap cells. Cell proliferation, invasion and apoptosis were determined by using CCK-8, Transwell and Flow cytometry assays, respectively. The target correlations between miR-30-5p and NORAD or RAB11A were confirmed by using dual luciferase reporter assay. Moreover, expression levels of RAB11A, the epithelial-mesenchymal transition (EMT) marker proteins and the Wnt pathway related proteins were measured by Western blotting. Tumor xenograft assay was used to study the effect of NORAD on tumor growth in vivo.

**Results:**

NORAD was upregulated in PC tissues and cells. Overexpression of NORAD promoted cell proliferation, invasion, EMT, and inhibited cell apoptosis; while knockdown of NORAD had the opposite effect. NORAD was found to be functioned as a ceRNA to bind and downregulated miR-30a-5p that was downregulated in PC tumor tissues. Rescue experiments revealed that miR-30a-5p could weaken the NORAD-mediated promoting effects on cell proliferation, invasion and EMT. Furthermore, RAB11A that belongs to a member of RAS oncogene family was verified as a target of miR-30a-5p, and reintroduction of RAB11A attenuated the effects of miR-30a-5p overexpression on cell proliferation, invasion, EMT and apoptosis of PC cells. More importantly, silencing RAB11A partially reversed the promoting effects of NORAD overexpression on cell proliferation, invasion and EMT of PC cells via the WNT/β-catenin pathway. Lastly, tumorigenicity assay in vivo demonstrated that NORAD increased tumor volume and weight via miR-30a-5p /RAB11A pathway.

**Conclusion:**

Our results indicated a significant role of NORAD in mechanisms associated with PC progression. NORAD promoted cell proliferation, invasion and EMT via the miR-30a-5p/RAB11A/WNT/β-catenin pathway, thus inducing PC tumor growth.

## Background

Prostate cancer (PC) is one of the most prevalent fatal tumors of the male genitourinary system and the second frequent cause of mortality in males worldwide [1, 2]. Despite numerous improvements had been made in the traditional diagnosis and treatment for patients with early-stage, the treatments for the advanced patients are still challenges [3]. Moreover, high metastatic rate of PC cells is a major cause of poor prognosis in prostate cancer patients [4]. Thus, finding novel molecular targets and new therapies for PC is necessary.

As a type of non-coding RNAs (ncRNAs), long non-coding RNAs (lncRNAs) are a class of RNAs with longer than 200 nucleotides and generally having no protein coding potentiality [5, 6], which can regulate the expression of downstream target genes through different mechanisms to function as carcinogens or suppressors in diverse tumors [7]. Some lncRNAs, such as FER1L4 [8], PlncRNA-1 [9], LEF1-AS1 [10], H19 [11] and HCG11 [12] had been identified to be involved in PC progression due to their abnormal expression. More importantly, lncRNAs can serve as competing endogenous RNAs (ceRNAs), which compete with miRNA for binding to target mRNAs, thus regulating miRNA availability targeted mRNAs. During the development of PC, the mechanism of ceRNA action of several lncRNA had been revealed. For example, lncRNA FOXC2-AS1 accelerated the tumor progression of prostate cancer cells by regulating the proliferation and tumor growth through miR-1253/EZH2 axis, thus leading to the poor prognosis of prostate cancer patients [13]. LncRNA UCA1 could function as a ceRNA to enhance PC progression via sponging miR143 followed by modulating the MYO6 expression [14], and miR-204 followed by modulating the CXCR4 expression [15]. LncRNA HCG11 suppressed cell progression by inhibiting miR-543-mediated PI3K/AKT signaling pathway in PC [16].

The lncRNA, noncoding RNA activated by DNA damage (NORAD) had been demonstrated to participate in various types of human carcinomas, such as lung cancer [17], osteosarcoma [18], epithelial ovarian cancer [19], in which lncRNAs functioned as ceRNAs and played critical roles in the promotion of cancers development. In addition, recent study demonstrated that NORAD was upregulated in PC cell lines, and knockdown of NORAD significantly suppressed PC cells proliferation, migration, and enhanced cell apoptosis [20]. However, the impact of NORAD on progression of prostate cancer and its possible mechanisms are still largely unknown. MiR-30a-5p was considered to be a tumor suppressor in several cancers, such as lung squamous cell carcinoma [21], colon cancer [22], non–small cell lung cancer [23], breast cancer [24] and gallbladder cancer [25]. Zhao et al. [26] revealed that miR-30a-5p was downregulated in PC patients, and in vitro experiments demonstrated that miR-30a-5p suppressed proliferation of PC cells by regulating PCLAF expression.

In the present study, NORAD was found to be significantly upregulated in PC tissues and cell lines, and overexpressed or silencing of NORAD could promote or suppress cell proliferation, invasion and EMT. Furthermore, bioinformatics analysis indicated that NORAD contained some complementary pairing with miR-30a-5p that was downregulated in PC tissues. Subsequently, we observed that NORAD enhanced cell proliferation, invasion and EMT of PC cells by sponging miR-30a-5p. Therefore, this study illustrates that NORAD-miR-30a-5p regulatory network plays critical role in the development of PC, which may provide a potential therapeutic target in PC patients.

## Materials and methods

### Tissue samples and cell culture

PC tumor tissues (n = 45) and adjacent normal tissues (n = 45) were obtained from PC patients from January 2017 to August 2018 in Huaihe Hospital of Henan University (Kaifeng, China). All collected tissues were immediately washed with sterile phosphate-buffered saline before being snap frozen in liquid nitrogen, and then stored at − 80 °C for further RNA and protein extraction. All patients in this study signed an informed consent form prior to the study, and all experiments gained the approval of the Ethics Committee for Clinical Experiments at Huaihe Hospital of Henan University.

Normal human prostate epithelial cell line (RWPE-1) and human PC cell lines (PC-3, LNCap, 22RV1, DU-145) were obtained from Shanghai Institute of Cell Biology (Shanghai, China). PC cell lines were maintained in RPMI-1640 medium supplemented with 10% fetal bovine serum (Sangon Biotech, Shanghai, China). RWPE-1 cells were cultured in DMEM containing 10% FBS (Sangon Biotech). All cells were kept at 37 °C in a humidified incubator with 5% CO_2_.

### Cell transfection

The gene-overexpression vectors (pcDNA-NORAD and pcDNA-RAB11A) and the control vector (pcDNA) were purchased from GenScript Biotech Corp. (Nanjing, China). The small interfering RNAs against NORAD (NORAD siRNA), RAB11A (RAB11A siRNA) and negative control siRNAs (NC siRNAs) were designed, synthesized and validated by Thermo Fisher Scientific (Waltham, MA, USA). MiR-30a-5p mimic, miR-30a-5p inhibitor and the corresponding negative control (NC mimic and NC inhibitor) were purchased from RiboBio (Guangzhou, China). All these plasmids and oligonucleotides were transfected into PC-3 or LNCap cells by using lipofectamine 2000 transfection reagent (Invitrogen, Carlsbad, USA) under the suggestion of the manufacturer. At 48 h after transfection, cells were harvested for further study.

### RNA extraction and quantitative polymerase chain reaction (qRT-PCR)

Total RNA was extracted from PC tissues and cells, and tumor tissues obtained from nude mice by using TRIzol Reagent (Invitrogen). Subsequently, a total of 1 μg RNA was reversed transcribed to cDNA by PrimeScript RT reagent Kit (Thermo Fisher Scientific, Waltham, MA, USA). The quantitative analysis of RAB11A mRNA and NORAD were performed by the SYBR ® Premix Ex Taq™ reagent (TaKaRa) and GAPDH was used as an internal control. The relative expression of miR-30a-5p was analyzed by SYBR PrimeScript miRNA RT‐PCR Kit (Takara Biotechnology, Dalian, China) with U6 as the internal control. qRT-PCR analysis was performed by CFX96 qPCR machine (Invitrogen, Carlsbad, CA, USA) with the following steps: an initial denaturation step at 95 °C for 3 min, followed by denaturation at 94 °C for 15 s, annealing at 55 °C for 25 s and extension at 72 °C for 15 s for 35 cycles. The quantification was calculated by the 2^−ΔΔCt^ method after normalized by internal control. The specific primer sequences used in our study were provided in Additional file 1: Table S1.

### Western blotting analysis

Proteins of tumor tissues from PC patients or nude mice, PC cells (PC-3 and LNCap) lysates were harvested with RIPA lysis buffer (Sangon Biotech, Shanghai, China) supplemented with protease inhibitor. Protein concentration was determined using the BCA protein assay. Then 25 μg of protein from each sample was separated by SDS-PAGE and transferred to PVDF membranes (Millipore, Billerica, MA, USA) in transfer buffer. After transfer, the membrane was blocked with 5% non-fat milk for 1 h at room temperature, and incubated with the following primary antibodies (BioTeke, Beijing, China) at 4 °C overnight: RAB11A (1:1000), N-cadherin (1:1000), E-cadherin (1:1000), Vimentin (1:1000), Snail (1:1000), β-catenin (1:1000), Cyclin D (1:1000), c-Myc (1:1000) and GAPDH (1:3000). Thereafter, the membranes were incubated with horseradish peroxidase-labelled secondary antibodies IgG (1:1000; BioTeke, Beijing, China) for 2 h at room temperature. Protein blots were visualized by using the ECL-Plus Western Blot Analysis Detection System (Thermo Fisher Scientific). GAPDH was used as an internal reference.

### Cell proliferation assay

Cell proliferation was assessed by using Cell Counting Kit-8 (Sigma, St. Louis, MO, USA). The transfected PC-3 and LNCap cells were plated into the 6-well plate at a density of 2 × 10^4^ cells/well. After incubation for 24, 48, 72 and 96 h, 10 μL CCK-8 solution was added into each well and cultured for additional 2 h in the darkness. Finally, cell proliferation was detected by measuring 450 nm absorbance. All experiments were repeated at least three times.

### Cell invasion assay

Transwell chamber (Millipore, Shijingshan, Haidian, USA) was applied for measuring cell invasion. Briefly, cells (0.5 mL; 2.5 × 10^4^ cells) in the upper chamber were resuspended in serum-free RPMI1640 medium, while RPMI1640 medium in the lower chamber was supplemented with 10% FBS. After 48 h of incubation at 37 °C, cells attached to the lower surface were fixed with 4% paraformaldehyde (Takara Biotechnology, Dalian, China) and stained with 0.3% crystal violet solution (Takara Biotechnology, Dalian, China). Then, the invasion numbers were determined by photographed with an inverted microscope.

### Flow cytometry analysis

After 48 h transfected with the above plasmids and oligonucleotides, PC-3 and LNCap cell apoptosis was detected by Annexin V-fluorescein isothiocyanate (FITC)/propidium iodide (PI) double staining using Annexin V-FITC Apoptosis Detection Kit (Thermo Fisher Scientific) for 15 min in darkness at room temperature. Then, the apoptosis rate was determined by flow cytometry using a CYTOMICS FC 500 flow cytometer (Beckman Coulter, USA).

### Dual luciferase reporter assays

Online bioinformatic tools StarBase 2.0 (http://starbase.sysu.edu.cn/) was used to predict the interactions of lncRNA NORAD and miR-30a-5p. The TargetScan (http://www.targetscan.org/vert_71/) was used to predict the binding sites within miR-30a-5p and RAB11A. Wild-type and mutant-type NORAD 3′-UTR that included the miR-30a-5p binding sites were subcloned into a pGL3 vector (Promega, Madison, WI) to create the WT-NORAD and MUT-NORAD luciferase reporter. Alternatively, the wild-type and mutant-type RAB11A 3′-UTR containing the miR-30a-5p binding sites were cloned into pGL3 vector to obtain the luciferase reporter vectors. Then, HEK-293 cells were transfected with miRNAs (NC mimic or miR-30a-5p mimic) along with the constructed luciferase reporter vectors by using Lipofectamine® 2000. After 48 h of treatment, the luciferase activity was determined by Multiskan FC Microplate Reader (Thermo Fisher Scientific, Waltham, MA, USA). Renilla signals were normalized to firefly signals. All experiments were performed at least three times.

### Tumor xenograft assay

In vivo tumor xenograft assay was performed by subcutaneously injecting the transfected PC-3 cells (1 × 10^6^) into the right flanks of BALB/c nude mice (weight, 22–24 g; age, 8 weeks; Shanghai SLAC Laboratory Animal Co., Ltd.). Briefly, the mice were randomly divided into four groups (NC siRNA, NORAD siARNA, NORAD siRNA + NC inhibitor, NORAD siRNA + miR-30a-5p inhibitor) according to the transfection. 5 mice were selected from each group and sacrificed to check the tumor volume weekly. After 4 weeks, all mice were sacrificed by intraperitoneal injection of sodium pentobarbital (150–200 mg/kg), and the tumor volume and weight were recorded. The tumor volume (mm^3^) was calculated with the formula (0.5 × Length × Width^2^). We stated that all animal experiments used in the present study were approved by the Experimental Animal Ethics Committee of Huaihe Hospital of Henan University (No. 00023).

### Statistical analysis

All statistical analysis was performed with SPSS version 22.0. Data were expressed as the mean ± standard error of the mean (SEM) of at least three experiments. Student's t test or one-way analysis of variance was applied for analyzing the data. *P* < 0.05 was considered statistically significant.

## Results

### NORAD expression is upregulated in PC tumor tissues and cell lines

**Fig. 1:**
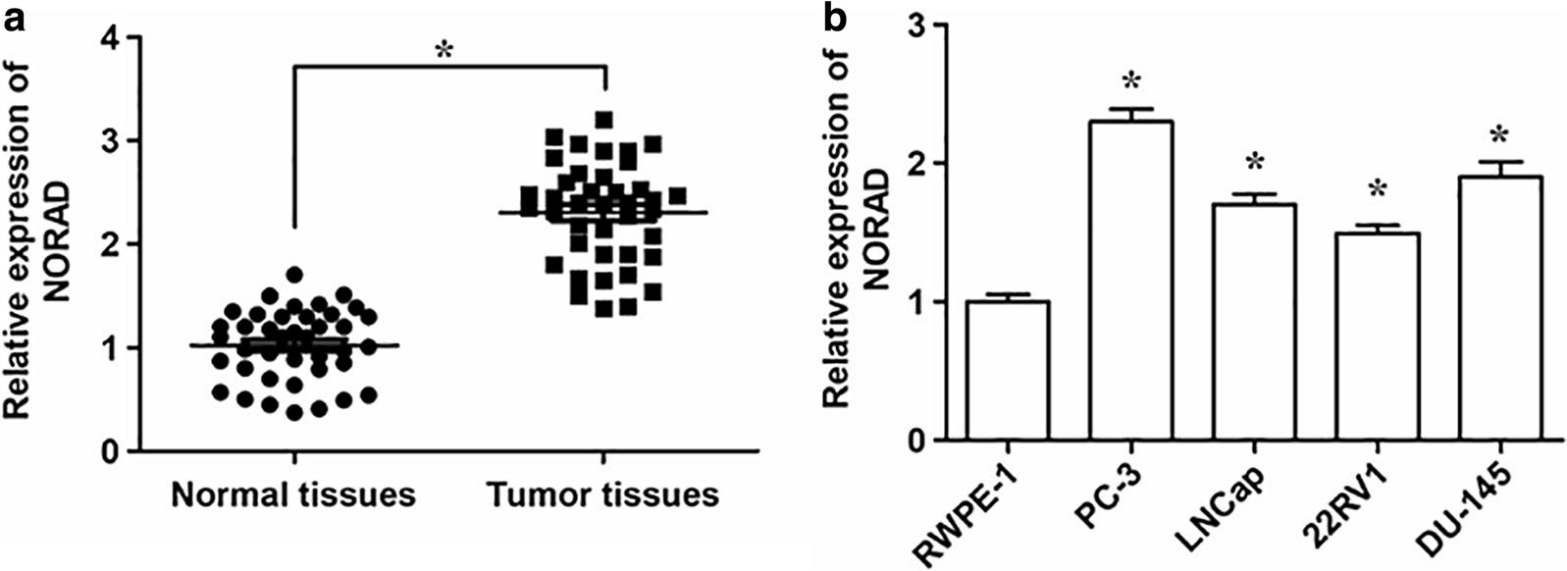
The expression level of LncRNA NORAD in PC tumor tissues and cell lines. **a** The relative expression levels of NORAD in PC tissues (Tumor tissues, n = 45) and adjacent normal tissues (Normal tissues, n = 45) determined by qRT-PCR. **b** Relative expression of NORAD was detected by qRT-PCR in PC cell lines (PC-3, LNCap, 22RV1, DU-145). The normal human prostate epithelial cell line (RWPE-1) served as a control. n = 3. The data were presented as the mean ± standard error of mean (SEM) and Student’s *t*-test was used for the comparison between 2 groups. *P < 0.05

The expression levels of NORAD in PC tumor tissues and adjacent normal tissues obtained from 45 PC patients were determined by qRT-PCR, and the expression level of NORAD in the tumor tissues group was remarkably higher than the normal group (Fig. 1a). In addition, qRT-PCR analysis also illustrated NORAD was significantly increased in PC cell lines (PC-3, LNCap, 22RV1, DU-145) compared with normal human prostate epithelial cell line (RWPE-1) (Fig. 1b). These results suggested that the abnormal expression of NORAD might be involved in PC progression.

### Overexpression of NORAD promotes PC cell proliferation, invasion and EMT process, and suppresses cell apoptosis

**Fig. 2:**
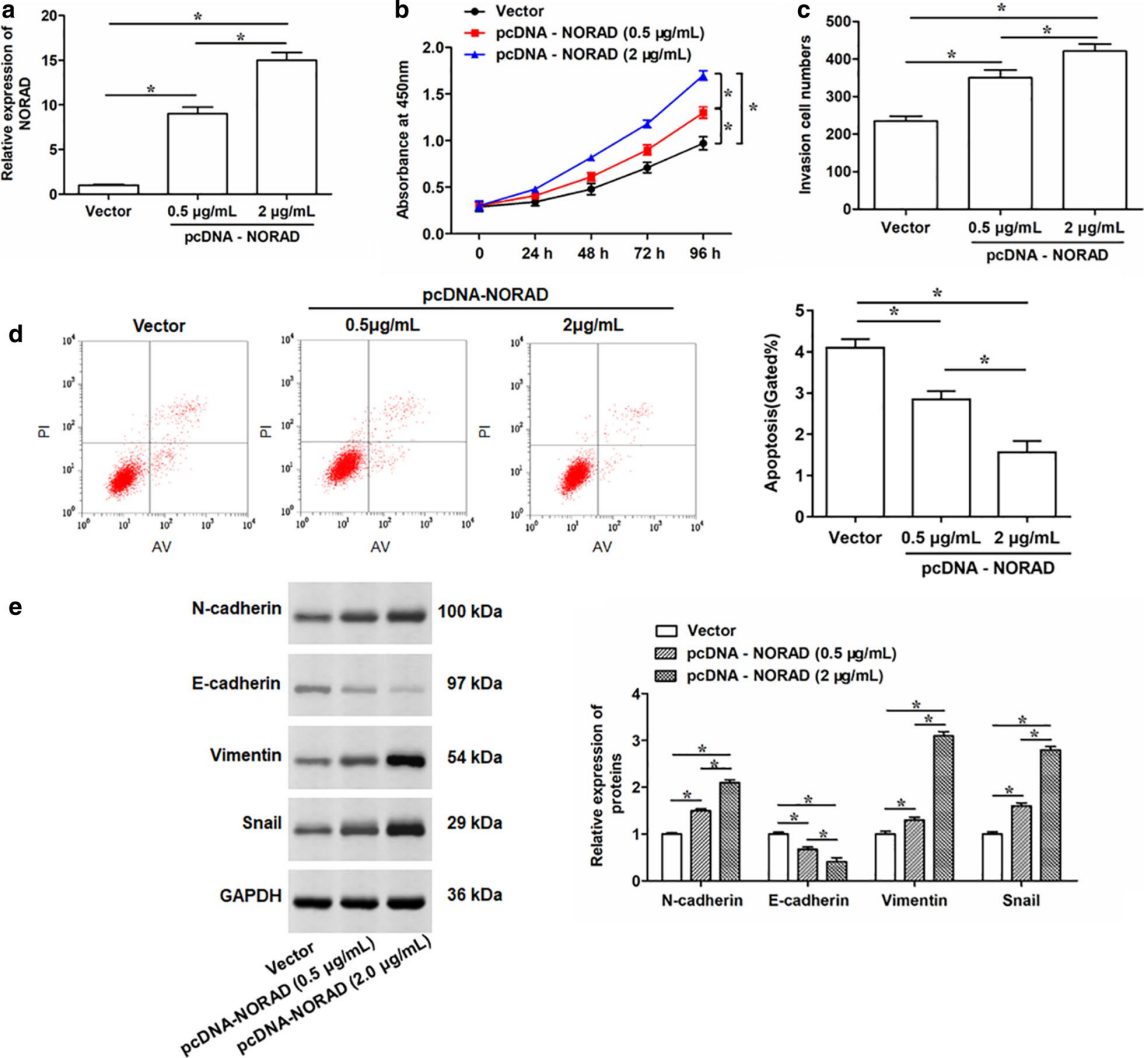
Effects of NORAD overexpression on cell proliferation, invasion, apoptosis, and EMT process of PC-3 cells. PC-3 cells were transfected with 0.5 or 2 μg/mL pcDNA-NORAD expression vector, or negative control Vector (0.5 μg/mL). **a** Expression of NORAD was then determined by qRT-PCR after 48 h transfection. **b** Cell proliferation of PC-3 cells after infection for 24, 48, 72 and 96 h were detected by CCK-8 assay. **c**, **d** Transwell and Flow cytometry was performed to determine cell invasion and apoptosis in PC-3 cells after infection for 48 h, respectively. **e** The expression levels of N-cadherin, E-cadherin, Vimentin and Snail in PC-3 cells after infection with pcDNA-NORAD for 48 h were detected by Western blotting. The data were presented as the mean ± standard error of mean (SEM), n = 3. Student’s *t*-test was used for the comparison between 2 groups, and one-way analysis of variance (ANOVA) was used for the comparison among more than 2 groups in this study. *P < 0.05

To further elucidate the mechanistic role of NORAD in PC, we transfected the PC-3 cells with empty vector or pcDNA NORAD overexpression vector (pcDNA-NORAD). Transfection with pcDNA-NORAD strikingly increased the expression of NORAD in PC-3 cells compared with vector group in a dose-dependent manner (Fig. 2a). The cell proliferation and invasion assay showed that overexpression of NORAD significantly increased the optical density and invasion cell numbers (Fig. 2b, c). While the apoptotic rate of PC-3 cell was decreased by NORAD overexpression (Fig. 2d). Subsequently, we further investigated the roles of NORAD overexpression on the EMT of PC-3 cells. As shown in Fig. 2e, the expression levels of N-cadherin, Vimentin and Snail were increased, and the expression level of E-cadherin was decreased in PC-3 cells with overexpression of NORAD (Fig. 2e). Besides that, the effects of NORAD overexpression on LNCap cell proliferation and invasion were also investigated. After elevating the expression of NORAD in LNCap cells using pcDNA-NORAD overexpression vector (pcDNA-NORAD) (Additional file 2: Fig. S1a), we observed that NORAD overexpression promoted the proliferation (Additional file 2: Fig. S1b) and invasion (Additional file 2:Fig. S1c) capacity of LNCap cells. These data demonstrated that NORAD could promote PC cell proliferation and invasion, inhibit cell apoptosis, and induce EMT process in a dose-dependent manner.

### Silencing of NORAD suppresses PC cell proliferation, invasion and EMT process, and enhances cell apoptosis

**Fig. 3:**
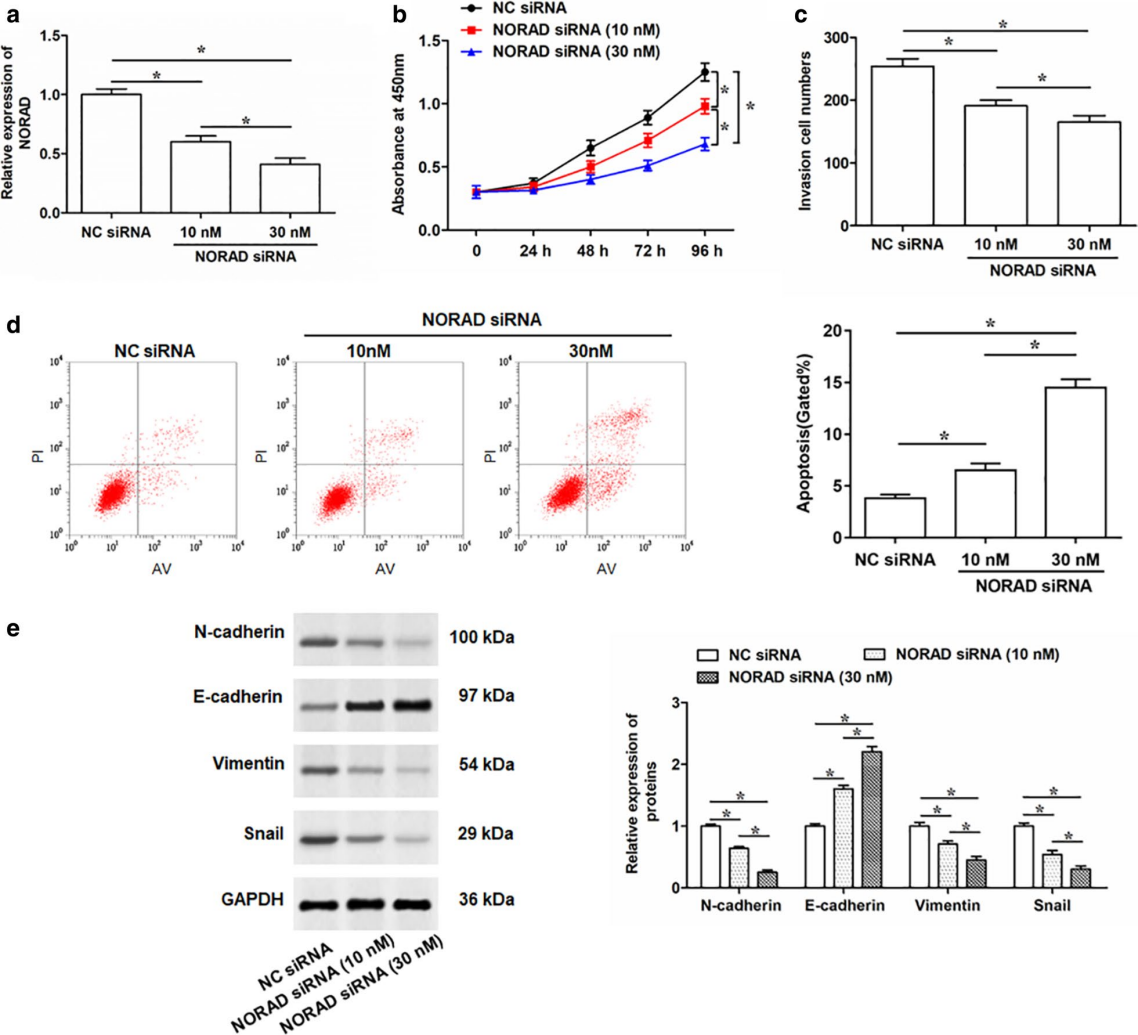
Effects of silencing NORAD on cell proliferation, invasion, apoptosis, and EMT process of PC-3 cells. PC-3 cells were transfected with 10 nM or 30 nM NORAD small interfering siRNA (NORAD siRNA), or negative control (10 nM). **a** Expression of NORAD was then determined by qRT-PCR after 48 h transfection. **b** Cell proliferation of PC-3 cells after infection for 24, 48, 72 and 96 h were detected by CCK-8 assay. **c**, **d** Transwell and Flow cytometry was performed to determine cell invasion and apoptosis in PC-3 cells after infection for 48 h, respectively. **e** The expression levels of N-cadherin, E-cadherin, Vimentin and Snail in PC-3 cells after transfection with NORAD siRNAs for 48 h were detected by Western blotting. The data were presented as the mean ± standard error of mean (SEM), n = 3. Student’s *t*-test was used for the comparison between 2 groups, and one-way analysis of variance (ANOVA) was used for the comparison among more than 2 groups in this study. *P < 0.05

Silence of NORAD was performed by transfecting NORAD siRNA into PC-3 cells. qRT-PCR assay suggested NORAD siRNA transfection remarkably suppressed the NORAD expression in PC-3 cells in a dose-dependent manner (Fig. 3a). Downregulation of NORAD significantly inhibited the cell proliferation and invasion compared with the negative control (Fig. 3b, c). The Flow cytometry assay demonstrated that knockdown of NORAD promoted cell apoptosis (Fig. 3d). In addition, silencing NORAD reduced the expression levels of N-cadherin, Vimentin and snail, and increased the expression level of E-cadherin (Fig. 3e). Besides that, transfection of NORAD siRNA into LNCap cells obviously decreased the expression of NORAD (Additional file 3: Fig. S2a). Then, we explored the role of NORAD silence in LNCap cell behaviors. The results showed that NORAD knockdown suppressed the proliferation (Additional file 3: Fig. S2b) and invasion (Additional file 3: Fig. S2c) capacity, and increased apoptosis of LNCap cells (Additional file 3: Fig. S2d). Therefore, these results indicated that knockdown of NORAD could suppress PC cell proliferation and invasion, promote cell apoptosis, and reduce EMT process.

### miR-30a-5p is a downstream target of NORAD, and partially abrogated the effects of NORAD overexpression on proliferation, invasion, apoptosis and EMT process of PC cells

**Fig. 4:**
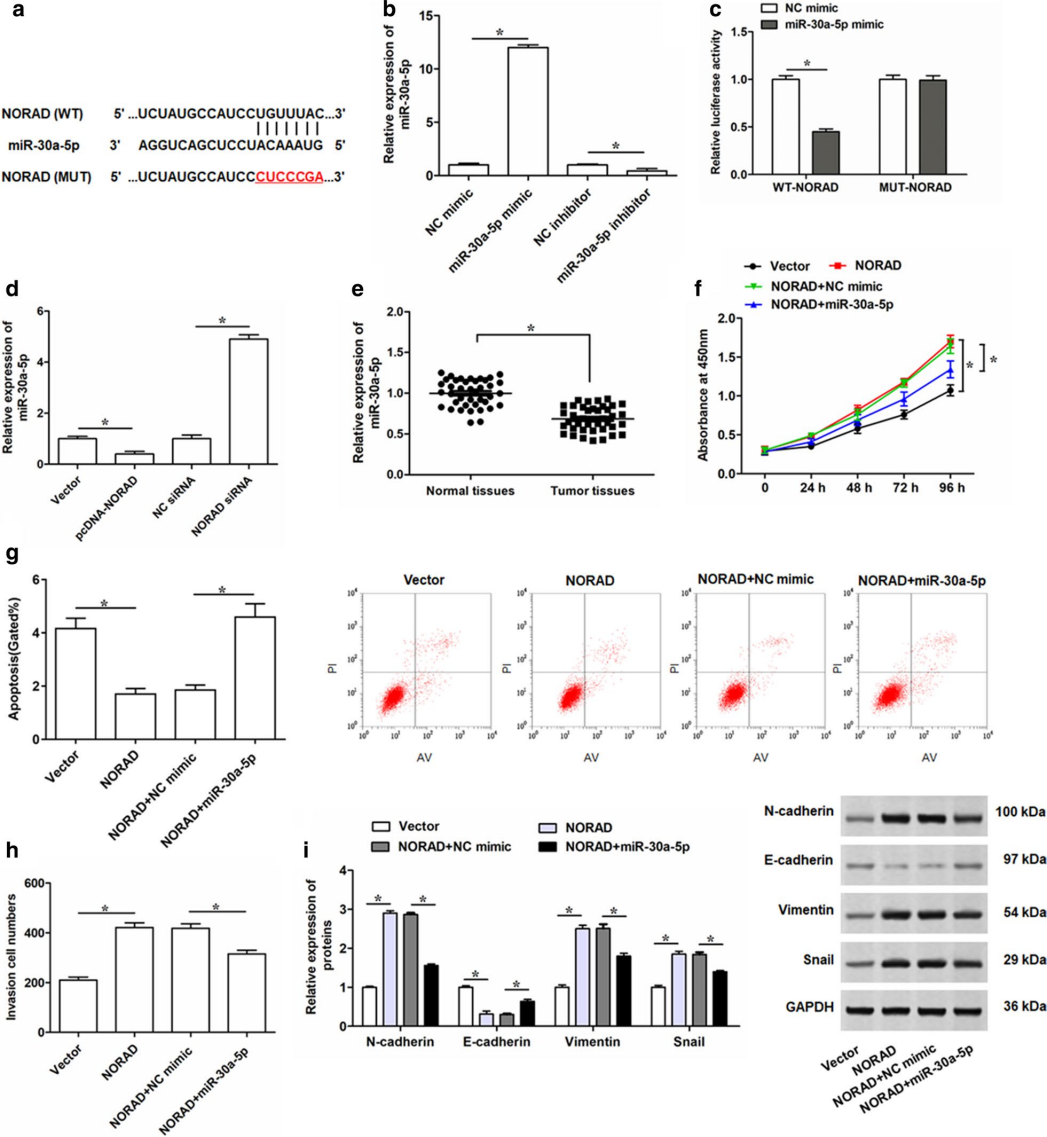
Reintroduction of miR-30a-5p weakens the effect of NORAD overexpression on cell proliferation, invasion, apoptosis and EMT process of PC-3 cells.** a** The potential binding sequences between NORAD and miR-30a-5p predicted by starBase. **b** qRT-PCR was conducted to determine the expression levels of miR-30a-5p in PC-3 cells transfected with 100 nM miR-30a-5p mimic, 100 nM miR-30a-5p inhibitor, or 100 nM their negative controls for 48 h. **c** Dual luciferase reporter assay was performed to determine the luciferase activity of HEK 293 T cells transfected with miR-30a-5p mimic and luciferase reporter vectors containing WT- or MUT- NORAD segment. **d** The expression level of miR-30a-5p was detected by qRT-PCR in PC-3 cell after being transfected with 2 μg/mL pcDNA-NORAD, 30 nM NORAD siRNA, or their negative controls for 48 h. **e** qRT-PCR was performed to determine the expression levels of miR-30a-5p in PC tissues (Tumor tissues, n = 45) and adjacent normal tissues (Normal tissues, n = 45) determined by qRT-PCR. Then, PC-3 cells were transfected with 2 μg/mL pcDNA-NORAD alone, or together with 100 nM miR-30a-5p mimic. **f** Cell proliferation of PC-3 cells after infection for 24, 48, 72 and 96 h were detected by CCK-8 assay. **g**, **h** After 48 h transfection, cell apoptosis and invasion was determined by Flow cytometry and Transwell, respectively. **i** The expression levels of N-cadherin, E-cadherin, Vimentin and Snail in PC-3 cells after infection of 48 h were detected by Western blotting. The data were presented as the mean ± standard error of mean (SEM). Student’s *t*-test was used for the comparison between 2 groups in this study. *P < 0.05

As we showed, NORAD played an important role in the proliferation, invasion, apoptosis and EMT process of PC cells. To further explore the role of NORAD in PC, we predicted the potential target miRNAs of NORAD by using the bioinformatics analysis. The results showed that miR-30a-5p possessed the binding sites with the 3′-UTR of NORAD sequence (Fig. 4a). We then constructed the luciferase vectors containing the wild-type and mutant (mutated bases were shown in red letters) binding sequences of NORAD (Fig. 4a). The overexpression or downregulation of miR-30a-5p were achieved through transfecting miR-30a-5p mimic or miR-30a-5p inhibitor into PC-3 cells, respectively (Fig. 4b). To confirm the interaction of miR-30a-5p with NORAD was mediated by the predicating binding sites, the dual luciferase reporter assay was conducted, and the results indicated that miR-30a-5p mimic obviously decreased the luciferase activity of WT-NORAD 3′-UTR reporter vectors, while no evident effect was found on luciferase activity of MUT-NORAD 3′-UTR reporter vectors (Fig. 4c). Then, we measured the effects of overexpressed and silencing NORAD on miR-30a-5p expression in PC-3 cells. The results illustrated that miR-30a-5p was suppressed by NORAD overexpression and increased by NORAD deficiency (Fig. 4d). In addition, qRT-PCR analysis also illustrated miR-30a-5p was significantly downregulated in PC tumor tissues (Fig. 4e). All these data indicated that NORAD interacted with miR-30a-5p and had an opposite pattern of expression.

Then, we further investigate the mechanism of NORAD interacting with miR-30a-5p in behaviors of PC cells by transfecting PC-3 cells with pcDNA-NORAD alone or together with miR-30a-5p mimic. Overexpression of NORAD remarkably promoted proliferation and invasion of PC-3 cells, while miR-30a-5p upregulation significantly attenuated these effects (Fig. 4f, h). In addition, miR-30a-5p rescued the suppressive effect of NORAD overexpression on apoptotic rate of PC-3 cells (Fig. 4g). Western blotting assays demonstrated that transfection of miR-30a-5p mimic strikingly reversed NORAD-induced increase in EMT process of PC-3 cells (Fig. 4i). In addition, miR-30a-5p was overexpressed or knocked down by transfecting miR-30a-5p mimic or miR-30a-5p inhibitor into LNCap cells, respectively (Additional file 4: Fig. S3a). Similar with the results of PC-3 cell behaviors, after transfection pcDNA-NORAD alone or together miR-30a-5p mimic into LNCap cells, we found that miR-30a-5p overexpression significantly reversed the effects of NORAD on LNCap cell proliferation, invasion and apoptosis (Additional file 4: Fig. S3b–d). Taken together, these results suggested NORAD may promote PC progression by regulating miR-30a-5p.

### miR-30a-5p inhibits PC progression by targeting RAB11A

**Fig. 5:**
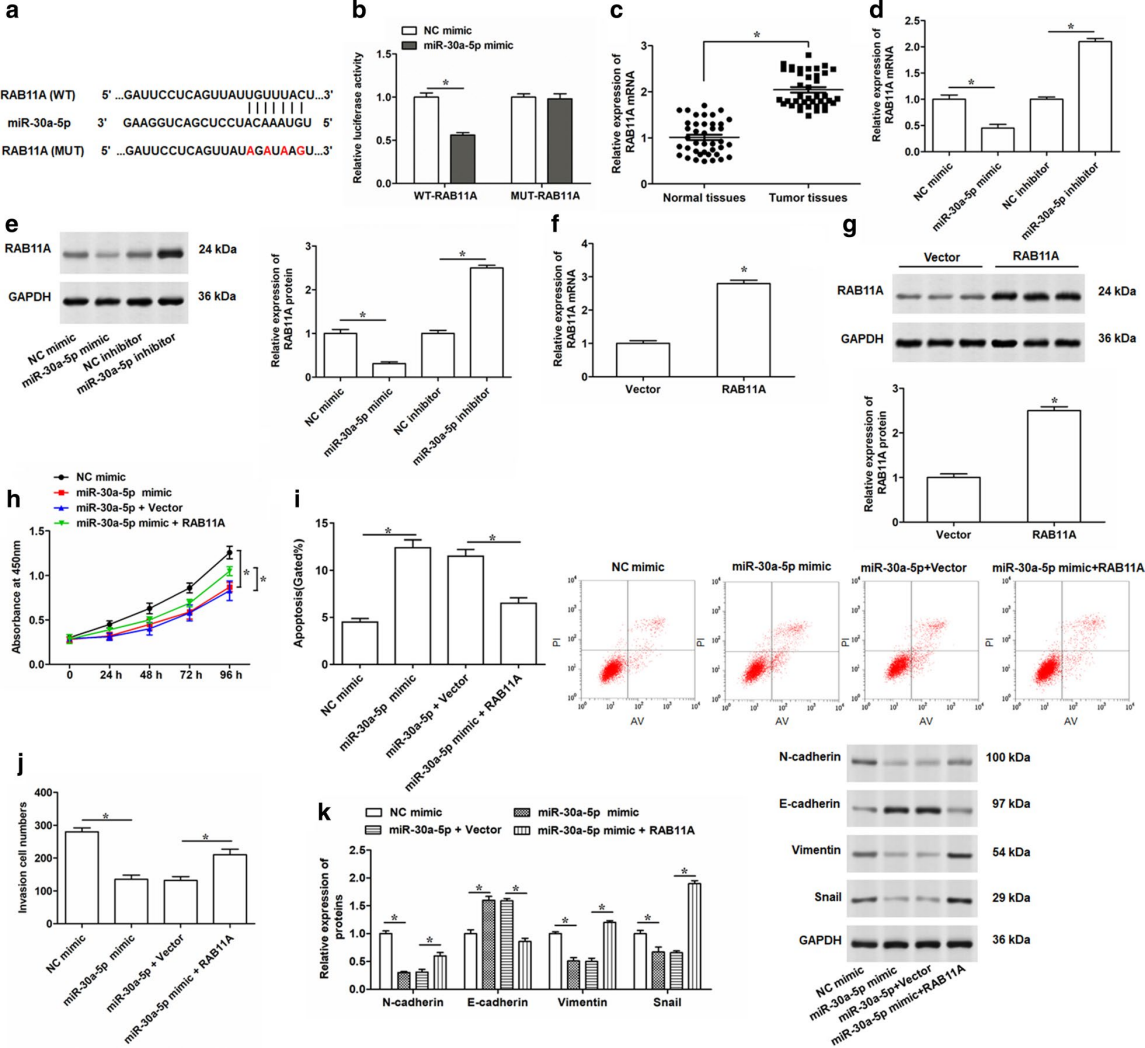
Effects of miR-30a-5p on cell proliferation, invasion, apoptosis and EMT in PC-3 cells by targeting RAB11A.** a** Diagram of the predicted miR-30a-5p binding sites in the 3′-UTR of RAB11A mRNA by TargetScan. **b** Dual luciferase reporter assay was performed to detect whether miR-30a-5p directly bound to the predicted binding sites in 3′-UTR of RAB11A mRNA. **c** The relative expression levels of RAB11A mRNA were determined by qRT-PCR in PC tissues (Tumor tissues, n = 45) and adjacent normal tissues (Normal tissues, n = 45) determined by qRT-PCR. **d**, **e** qRT-PCR and Western blotting assays were conducted to determine the mRNA and protein expression levels of RAB11A in PC-3 cells transfected with 100 nM miR-30a-5p mimic, 100 nM miR-30a-5p inhibitor, or 100 nM their negative controls for 48 h. **f**, **g** After infection PC-3 cells with RAB11A overexpression vector (1.5 μg/mL) for 48 h, the expression of RAB11A was determined by qRT-PCR and Western blotting at mRNA and protein levels. Then, PC-3 cells were transfected with 100 nM miR-30a-5p mimic alone, or together with 1.5 μg/mL RAB11A overexpression vector. **h** CCK-8 was used to detect the cell proliferation of PC-3 cells after transfectio for 48 h. **i, j** Flow cytometry and Transwell was performed to determined apoptosis and invasion in PC-3 cells after 48 h transfection. **k** The expression levels of N-cadherin, E-cadherin, Vimentin and Snail were detected by Western blotting in transfected PC-3 cells. The data were presented as the mean ± standard error of mean (SEM). Student’s t test was used for the comparison between 2 groups in this study. *P < 0.05

To further detect the effect of miR-30a-5p which was a target of NORAD on the development of PC cells, we predicted the downstream targets of miR-30a-5p by online bioinformatic tool and found that miR-30a-5p could bind to the 3′-UTR of RAB11A (Fig. 5a). The dual luciferase reporter assay indicated the luciferase activity of the WT-RAB11A 3′-UTR remarkably decreased after transfecting with miR-30a-5p mimic. While the luciferase activity displayed no significant changes in luciferase reporter vector containing MUT-RAB11A 3′-UTR compared the control group (Fig. 5b). Then, we measured the expression of RAB11A mRNA in PC tumor tissues and adjacent normal tissues, and the results showed that RAB11A mRNA expression was notably upregulated in PC tumor tissues (Fig. 5c). Overexpression of miR-30a-5p suppressed the expression of RAB11A at mRNA and protein levels; while knockdown of miR-30a-5p increased the mRNA and protein levels of RAB11A in PC-3 cells (Fig. 5d, e), suggesting an opposite expression pattern of miR-30a-5p and RAB11A in PC-3 cells. Overexpression of RAB11A were achieved by transfecting PC-3 cell with RAB11A overexpression vector (Fig. 5f, g). Subsequently, CCK-8 and Transwell assays illustrated that overexpressed miR-30a-5p inhibited proliferation and invasion of PC-3 cells, while reintroduction of RAB11A could weakened these suppressive effects (Fig. 5h, j). Apoptotic rate was strikingly increased by miR-30a-5p overexpression in PC-3 cells, which was remarkably eliminated by RAB11A regaining (Fig. 5i). In addition, overexpression of miR-30a-5p resulted in an evident decrease in expression of N-cadherin, Vimentin and Snail, and increase in E-cadherin expression, which were reversed by co-transfecting with miR-30a-5p and RAB11A (Fig. 5k). The transfection efficiencies of RAB11A overexpression vector in LNCap cells were determined (Additional file 5: Fig. S4a, b). Besides that, LNCap cells were transfected with miR-30a-5p mimic alone or together with RAB11A overexpression vector, and we found that the inhibitory effects of miR-30a-5p overexpression on LNCap cell proliferation and invasion, as well as the promoting effect on cell apoptosis, were weakened by the reintroduction of RAB11A (Additional file 5: Fig. S4c–e). This evidence suggested that miR-30a-5p inhibited PC cell proliferation, invasion and EMT processes by targeting RAB11A.

### NORAD regulates cell proliferation, invasion, apoptosis and EMT process of PC cells by miR-30a-5p/RAB11A/WNT/β-catenin pathway

**Fig. 6:**
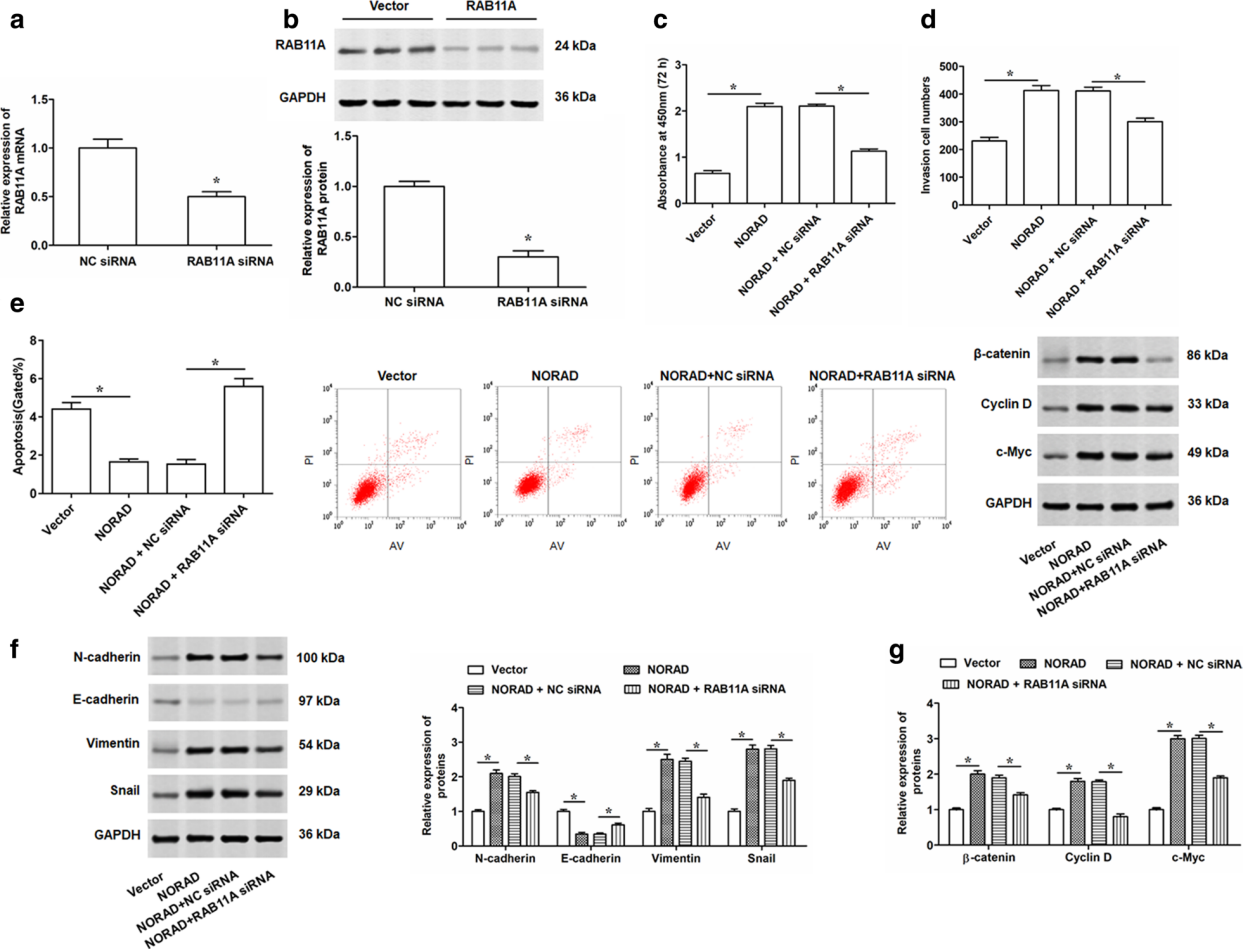
NORAD promotes cell proliferation, invasion and EMT, suppresses cell apoptosis via miR-30a-5p/RAB11A/WNT/β-catenin pathway in PC-3 cells. PC-3 cells were transfected with 50 nM RAB11A siRNA for 48 h. **a**,** b** Interference efficiencies of RAB11A siRNA were determined by qRT-PCR and Western blotting, respectively. Subsequently, PC-3 cells were transfected with 2 μg/mL pcDNA-NORAD alone, or together with 50 nM RAB11A siRNA. **c** After 48 h transfection, CCK-8 was used to detect the cell proliferation. **d**, **e** Cell invasion and apoptosis was determined by Transwell and Flow cytometry in PC-3 cells after 48 h transfection with NORAD alone or together with RAB11A siRNA. **f**, **g** Western blotting was used to determine the expression levels of related proteins of EMT (N-cadherin, E-cadherin, Vimentin and Snail) and WNT pathway (β-catenin, Cyclin D and c-Myc) in PC-3 cells after 48 h transfection. The data were presented as the mean ± standard error of mean (SEM), n = 3. Student’s *t*-test was used for the comparison between 2 groups in this study. *P < 0.05

Then, to explore whether NORAD affected the behaviors of PC-3 cells by RAB11A-mediated WNT pathway, we performed the rescue experiments by transfecting PC-3 cells with NORAD alone or together with RAB11A siRNA. qRT-PCR and Western blotting were conducted to detect the downregulation of RAB11A in PC-3 cells after being transfected with RAB11A siRNA (Fig. 6a, b). Further, cell proliferation and invasion assays illustrated that silencing RAB11A could weaken the promoting effects of NORAD overexpression on PC-3 cells (Fig. 6c, d). Similarly, overexpression of NORAD suppressed the cell apoptosis, while co-transfecting with RAB11A siRNA reversed this effect (Fig. 6e). Western blotting results demonstrated that the EMT process was enhanced by the overexpression of NORAD, but downregulation of RAB11A attenuated the improving effect of NORAD on the EMT in PC-3 cells (Fig. 6f). Furthermore, reintroduction of RAB11A could reverse NORAD-mediated promoting effects on the expression of β-catenin, cyclin D and c-Myc proteins (Fig. 6f). The expression of RAB11A in LNCap cells were also decreased by the transfection of RAB11A siRNA (Additional file 6: Fig. S5a, b). Then, LNCap cells were transfected with NORAD alone or together with RAB11A siRNA, and the results showed that RAB11A knockdown obviously reversed the promoting effects on LNCap cell proliferation, invasion and EMT, and the suppressive effects on cell apoptosis (Additional file 6: Fig. S5c–f). Moreover, the positive effect of NORAD on the activation of WNT pathway was weakened by reintroduction of RAB11A in LNCap cells (Additional file 6: Fig. S5g). All these results suggested NORAD might affect the behaviors of PC cells by regulating RAB11A. In above experiments, we demonstrated the NORAD had important effects on cell proliferation, invasion, apoptosis and EMT process of PC cells. Therefore, our results suggested NORAD may be involved in regulating PC progression via miR-30a-5p/RAB11A /WNT/β-catenin pathway.

### Downregulation of NORAD inhibits in vivo tumor growth, but miR-30a-5p inhibitor weakens this suppressive effect

**Fig. 7:**
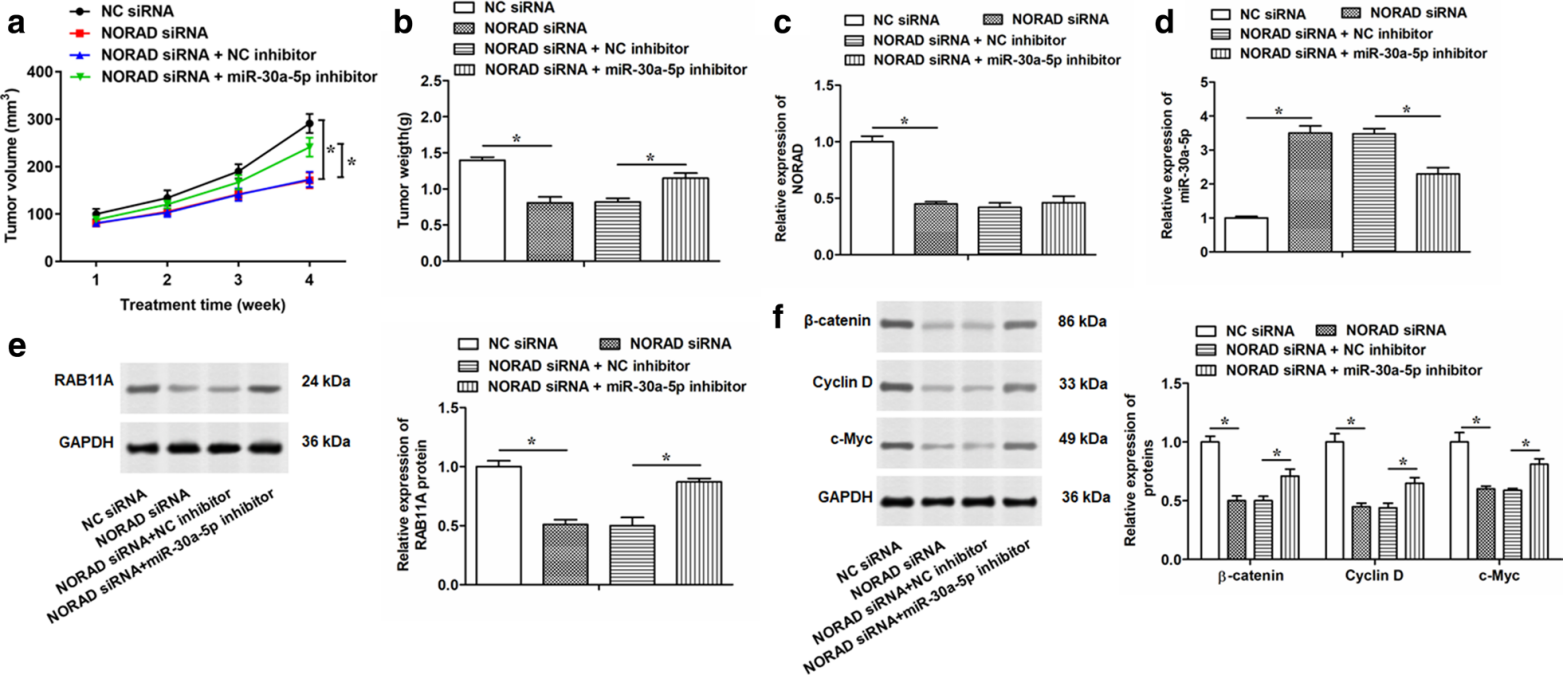
Downregulation of NORAD suppresses tumor growth in vivo. PC-3 cells were transfected with NORAD siRNA alone, or together with miR-30a-5p inhibitor for 48 h. Then, the transfected PC-3 cells were subcutaneously injected into the right flanks of nude mice. **a** The tumor volume was measured once a week for four weeks and calculated by the formula of 0.5 × Length × Width^2^. **b** Tumor-bearing mice were killed after four weeks’ cultivation, and the weight of the tumor was weighed in each group. **c**, **d** The expression levels of NORAD and miR-30a-5p were determined by qRT-PCR in tumor tissues harvested from the nude mice in each group. **e, f** Western blotting was used to detect the expression of RAB11A protein and the related proteins of WNT pathway (β-catenin, Cyclin D and c-Myc) in tumor tissues harvested from the nude mice in each group. The total number of mice is eighty (n = 5/group). The data were presented as the mean ± standard error of mean (SEM). Student’s *t*-test was used for the comparison between 2 groups in this study. *P < 0.05

Finally, to investigate the tumorigenic effects of NORAD on BALB/C nude mice, we conducted a xenograft mouse model by subcutaneous injection of the PC-3 cells transfected with NORAD siRNA alone or together with miR-30a-5p inhibitor. Then, tumor volumes were measured weekly, and tumor weight were detected at the fourth week. The results showed that the mice with NORAD siRNA infected cells significantly inhibited tumor volume (Fig. 7a) and tumor weight (Fig. 7b), while miR-30a-5p inhibitor obviously decreased the suppressive effect of NORAD on the tumor growth. Moreover, we detected the expression levels of NORAD and miR-30a-5p in the tumors obtained from the mice subcutaneously injected with the transfected cells, and the results indicated NORAD was downregulated and miR-30a-5p was increased in the tumor tissues of nude mice in each group (Fig. 7c, d). Co-transfecting with NORAD siRNA and miR-30a-5p inhibitor had no effect on the NORAD expression, but decreased the expression of miR-30a-5p (Fig. 7c, d). The expression of RAB11A was decreased in the tumor tissues with injection of PC-3 cells transfected with NORAD siRNA, and miR-30a-5p inhibitor weakened this result (Fig. 7e). Similarly, Western blotting assay suggested miR-30a-5p inhibitor partially eliminated the suppressive effect of NORAD siRNA on the WNT pathway, thus inhibiting the tumor growth (Fig. 7f). In conclusion, these data supported that NORAD play a role in promoting in vivo tumor growth by suppressing the expression of miR-30a-5p.

## Discussion

Increasing evidence suggest that lncRNAs can play oncogenic or anti-cancer roles in the pathogenesis of PC [27]. In our study, the expression level of NORAD was significantly upregulated in the PC tumor tissues compared with adjacent normal tissues. Our results also illustrated that overexpression of NORAD promoted PC cell proliferation, invasion and EMT process, and inhibited cell apoptosis; while silencing NORAD had the opposite effects on the behaviors of PC cells. Then, further explorations demonstrated that NORAD regulated the cell proliferation, invasion and EMT via the miR-30a-5p/RAB11A pathway.

Previous studies had demonstrated that NORAD played important roles in various types of cancer. He et al. found that NORAD was upregulated in papillary thyroid carcinoma, and promoted PTC cell growth, invasion and migration by suppressing miR-202-5p expression [28]. Upregulation of NORAD promoted gastric cancer progression by activating the RhoA/ROCK1 axis, which correlated with poorer prognosis [29]. Zhou et al. found that high expression of NORAD promoted breast cancer progression through regulating TGF-β pathway and had a poor prognosis [30]. Moreover, upregulation of NORAD was demonstrated to affect the progression of diabetic nephropathy through targeting miR-520 h to increase the expression of TLR4 [31]. What's more, NORAD had been demonstrated to enhance the proliferation and migration of PC cells and suppressed their apoptosis in PC [20]. Consistently, our results showed that overexpression of NORAD could promote PC cell proliferation and invasion, inhibit cell apoptosis, and induce EMT; while knockdown of NORAD reversed these results. Taken together, these data indicated that NORAD had promoting effects on cell proliferation, invasion and EMT in PC cells. In other words, NORAD functioned as an oncogene in the development of PC.

As we all know, lncRNAs can act as the microRNA decoys to sequester miRNAs, thus favoring expression of repressed target mRNAs, or lncRNAs can suppress the expression of target mRNAs by competitively binding to miRNAs [32]. Several studies had proved that NORAD serving as a ceRNAs could bind to miRNA, thus being involved in cellular processes in cancers, including PC [13, 18, 19]. In present study, bioinformatics prediction revealed that NORAD could interact with several miRNAs, and we selected miR-30a-5p for further investigations owing to its role in the regulation of cell behaviors in several types of cancers. MiR-30a-5p was significantly decreased in osteosarcoma, and inhibited cell proliferation, migration and tumor growth in vivo [33]. Li et al. reported that miR-30a-5p suppressed breast tumor growth and metastasis through inhibition of LDHA-mediated Warburg effect [24]. In addition, miR-30a-5p functioned as an anti-oncogene to inhibit the growth of renal cell carcinoma by modulating the expression of GRP78 [34]. In our results, miR-30a-5p attenuated the effects of NORAD overexpression on cell proliferation, invasion and EMT process of PC cells, suggesting that NORAD might function as a ceRNA to bind miR-30a-5p to enhance PC progression.

The miRNAs play important roles in regulating the gene expression via binding to the 3′-UTR of targeted genes. RAB11A, as one of the targeted genes of miR-30a-5p, was further predicted by using the online bioinformatics tool TargetScan and verified by luciferase reporter assays. RAB11A belonging to the Rab family of the small GTPase superfamily is reported to be involved in many cellular processes including protein secretion, phagocytosis, signal transmission and cell migration [35]. Previous data had shown RAB11A was upregulated in thyroid cancer [36], breast cancer [37, 38], pancreatic cancer [39], and bladder cancer [40], suggesting the importance of RAB11A in human cancers’ development. In our study, we found that RAB11A expression was upregulated and had an opposite expression pattern with miR-30a-5p in PC tumor tissues. Furthermore, the functional experiments revealed that the overexpression of miR-30a-5p inhibited the PC cell proliferation, invasion and EMT process, while reintroduction of RAB11A partially weakened suppressive effects of miR-30a-5p overexpression, which suggested that miR-30a-5p inhibited PC tumor development through targeting RAB11A. In addition, our results showed silencing RAB11A also attenuated the promoting effects of NORAD overexpression on cell proliferation, invasion and EMT process of PC cells. WNT/β-catenin signaling is known to be involved in a broad range of cellular processes, such as cell proliferation, cell invasion, and cell differentiation [41]. Yu et al. had reported that RAB11A promoted cell growth, invasion, and cell cycle progression by activating GSK3β/WNT/β-catenin signaling pathway in pancreatic cancer [39]. Moreover, RAB11A was upregulated by lncRNA SNHG1 and activated the WNT/β-catenin pathway in invasive pituitary tumors [42]. In the present study, silencing RAB11A partially reversed the promoting effects of overexpression NORAD on the expression levels of WNT target genes including β-catenin, Cyclin D and c-Myc. Taken together, these results suggested NORAD promote PC development through NORAD/miR-30a-5p/RAB11A /WNT/β-catenin pathway.

## Conclusion

In summary, our results demonstrated that the expression of NORAD was significantly upregulated and promoted PC cell proliferation, invasion and EMT process via targeting miR-30a-5p/RAB11A/WNT/β-catenin pathway. These findings may help us to better understand the regulatory mechanisms of NORAD in PC, thus providing a potential target for the treatment of PC patients.

## Supplementary information

**Additional file 1: Table S1.** Primer sequences of related genes for reverse transcription quantitative polymerase chain reaction.

**Additional file 2: Figure S1.** Effects of NORAD overexpression on cell proliferation and invasion in LNCap cells.

**Additional file 3: Figure S2.** Effects of silencing NORAD on cell proliferation, invasion and apoptosis in LNCap cells.

**Additional file 4: Figure S3.** Reintroduction of miR-30a-5p weakens the effect of NORAD overexpression on cell proliferation, invasion and apoptosis in LNCap cells.

**Additional file 5: Figure S4.** Effects of miR-30a-5p on cell proliferation, invasion and apoptosis in LNCap cells by targeting RAB11A.

**Additional file 6: Figure S5.** NORAD promotes cell proliferation, invasion and EMT, suppresses cell apoptosis via miR-30a-5p/RAB11A/WNT/β-catenin pathway in LNCap cells.

## Data Availability

Not applicable.

## Abbreviations

PC: Prostatic cancer
lncRNA: Long noncoding RNA
NORAD: Noncoding RNA activated by DNA damage
RAB11A: Belonging to the Rab family of the small GTPase superfamily
eRNAs: Competing endogenous RNAs
EMT: Epithelial-mesenchymal transition
3′-UTR: 3′-Untranslatedregion
RWPE-1: Normal human prostate epithelial cell line
