# Supplementary material for: Long non-coding RNA NORAD contributes to the proliferation, invasion and EMT progression of prostate cancer via the miR-30a-5p/RAB11A/WNT/β-catenin pathway
Source: Cancer Cell Int. 2020 Nov 27;20:571. doi: 10.1186/s12935-020-01665-2 (PMC7694907; doi:10.1186/s12935-020-01665-2)
Supplement: Supplementary file 1 — Additional file 1: Table S1. Primer sequences of related genes for reverse transcription quantitative polymerase chain reaction. [file 12935_2020_1665_MOESM1_ESM.docx]

**Additional file 1: Table S1. Primer sequences of related genes for reverse transcription quantitative polymerase chain reaction**

| Gene | Primer sequence (5’-3’) |
| --- | --- |
| NORAD | Forward: TGATAGGATACATCTTGGACATGGA  Reverse: AACCTAATGAACAAGTCCTGACATACA |
| MiR-30a-5p | Forward: GGGCCTGTAAACATCCTCG  Reverse: GAATACCTCGGACCCTGC |
| RAB11A | Forward: AAAGCAAGAGCACCATTGGAG  Reverse: TGCCCTGCTGTGTCCCAT |
| GAPDH | Forward: GGAGCGAGATCCCTCCAAAAT  Reverse: GGCTGTTGTCATACTTCTCATGG |
| U6 | Forward: CTCGCTTCGGCAGCACA  Reverse: AACGCTTCACGAATTTGCGT |
