# Supplementary material for: Long non-coding RNA NORAD contributes to the proliferation, invasion and EMT progression of prostate cancer via the miR-30a-5p/RAB11A/WNT/β-catenin pathway
Source: Cancer Cell Int. 2020 Nov 27;20:571. doi: 10.1186/s12935-020-01665-2 (PMC7694907; doi:10.1186/s12935-020-01665-2)

**Additional file 2: Figure S1. Effects of NORAD overexpression on cell proliferation and invasion in LNCap cells.** LNCap cells were transfected with 0.5 μg/mL, 2 μg/mL pcDNA-NORAD expression vector or negative control Vector (0.5 μg/mL). **a** Expression of NORAD was determined by qRT-PCR after 48 h transfection. **b** Cell proliferation of LNCap cells after infection for 24 h, 48 h, 72 h and 96 h were detected by CCK-8 assay. **c** Transwell assay was performed to determine cell invasion in LNCap cells after infection for 48 h, respectively. The data were presented as the mean ± standard error of mean (SEM), n=3. Student’s t test was used for the comparison between 2 groups, and one-way analysis of variance (ANOVA) was used for the comparison among more than 2 groups in this study. * *P* < 0.05


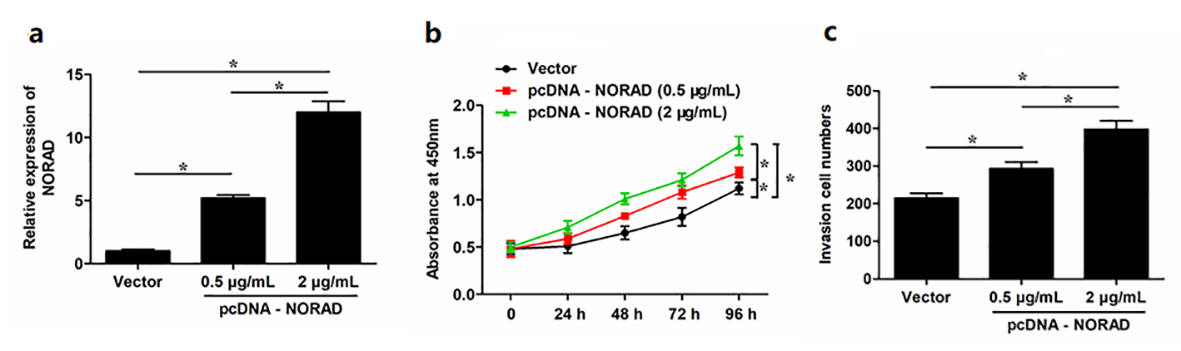

Supplement: Supplementary file 2 — Additional file 2: Figure S1. Effects of NORAD overexpression on cell proliferation and invasion in LNCap cells. [file 12935_2020_1665_MOESM2_ESM.docx]
