# Supplementary material for: Long non-coding RNA NORAD contributes to the proliferation, invasion and EMT progression of prostate cancer via the miR-30a-5p/RAB11A/WNT/β-catenin pathway
Source: Cancer Cell Int. 2020 Nov 27;20:571. doi: 10.1186/s12935-020-01665-2 (PMC7694907; doi:10.1186/s12935-020-01665-2)

**Additional file 4: Figure S3.** **Reintroduction of miR-30a-5p weakens the effect of NORAD overexpression on** **cell proliferation, invasion and apoptosis in LNCap cells. a** qRT-PCR was conducted to determine the expression levels of miR-30a-5p in LNCap cells transfected with 100 nM miR-30a-5p mimic, 100 nM miR-30a-5p inhibitor, or 100 nM their negative controls for 48 h. Then, LNCap cells were transfected with 2 μg/mL pcDNA-NORAD alone or together with 100 nM miR-30a-5p mimic. **b** Cell proliferation was detected by CCK-8 assay in LNCap cells after infection for 24 h, 48 h, 72 h and 96 h. **c, d** After 48 h transfection, cell invasion and apoptosis was determined by Transwell and Flow cytometry assay, respectively. The data were presented as the mean ± standard error of mean (SEM). Student’s t test was used for the comparison between 2 groups in this study. * *P* < 0.05


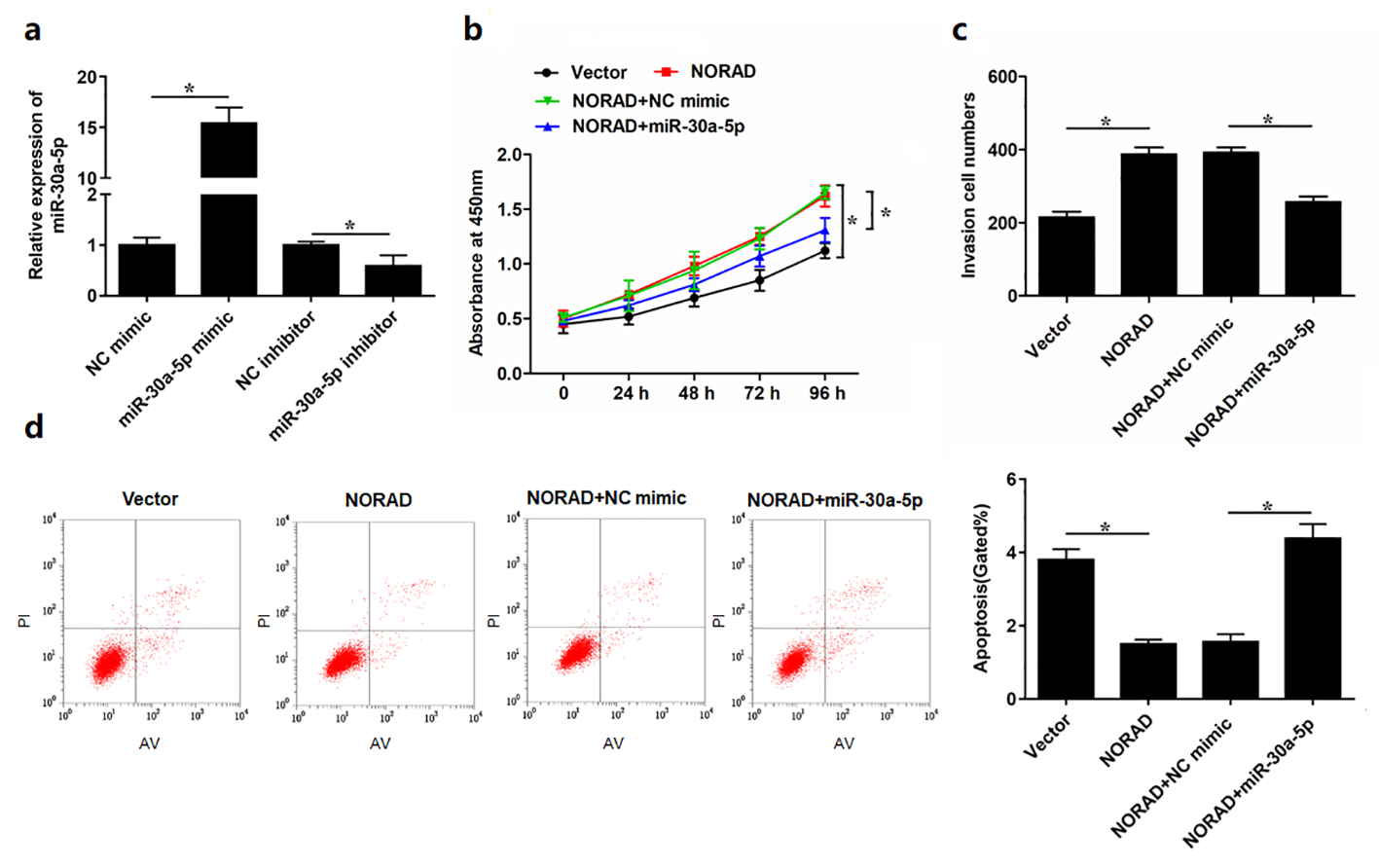

Supplement: Supplementary file 4 — Additional file 4: Figure S3. Reintroduction of miR-30a-5p weakens the effect of NORAD overexpression on cell proliferation, invasion and apoptosis in LNCap cells. [file 12935_2020_1665_MOESM4_ESM.docx]
