# Supplementary material for: Long non-coding RNA NORAD contributes to the proliferation, invasion and EMT progression of prostate cancer via the miR-30a-5p/RAB11A/WNT/β-catenin pathway
Source: Cancer Cell Int. 2020 Nov 27;20:571. doi: 10.1186/s12935-020-01665-2 (PMC7694907; doi:10.1186/s12935-020-01665-2)

**Additional file 5: Figure S4. Effects of miR-30a-5p on cell proliferation, invasion and apoptosis in LNCap cells by targeting RAB11A. a, b** After infection LNCap cells with RAB11A overexpression vector (1.5 μg/mL) for 48 h, the expression of RAB11A was determined by qRT-PCR and Western blotting at mRNA and protein levels. Then, LNCap cells were transfected with 100 nM miR-30a-5p mimic alone, or together with 1.5 μg/mL RAB11A overexpression vector. **c** CCK-8 was used to detect the cell proliferation of LNCap cells after transfection for 48 h. **d, e** Transwell and Flow cytometry was performed to determined invasion and apoptosis in LNCap cells after 48 h transfection, respectively. The data were presented as the mean ± standard error of mean (SEM). Student’s t test was used for the comparison between 2 groups in this study. * *P*< 0.05


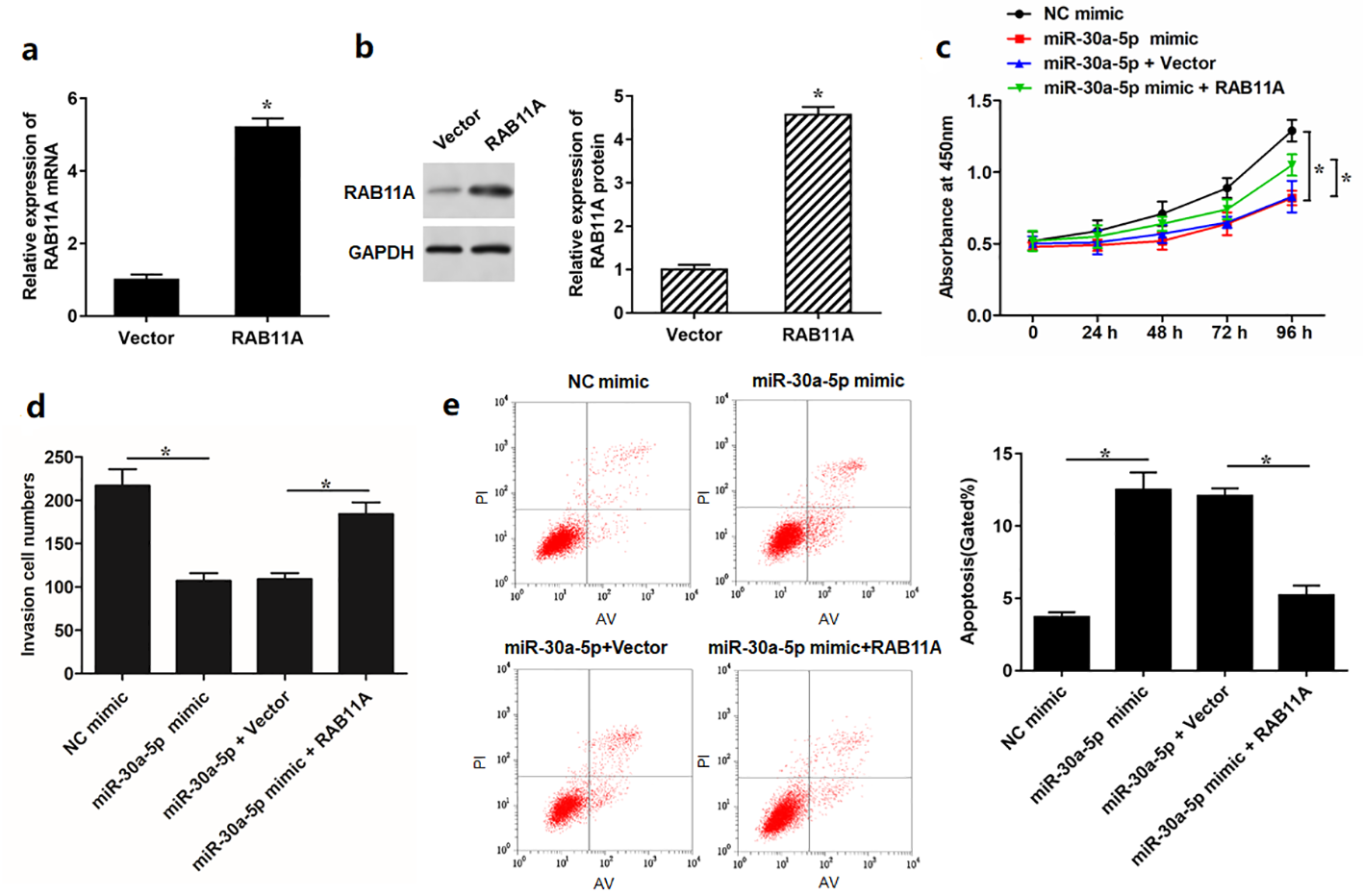

Supplement: Supplementary file 5 — Additional file 5: Figure S4. Effects of miR-30a-5p on cell proliferation, invasion and apoptosis in LNCap cells by targeting RAB11A. [file 12935_2020_1665_MOESM5_ESM.docx]
