# Supplementary material for: Long non-coding RNA NORAD contributes to the proliferation, invasion and EMT progression of prostate cancer via the miR-30a-5p/RAB11A/WNT/β-catenin pathway
Source: Cancer Cell Int. 2020 Nov 27;20:571. doi: 10.1186/s12935-020-01665-2 (PMC7694907; doi:10.1186/s12935-020-01665-2)

**Additional file 6: Figure S5.** **NORAD promotes cell proliferation, invasion and EMT, suppresses cell apoptosis via miR-30a-5p/RAB11A/WNT/β-catenin pathway in LNCap cells.** LNCap cells were transfected with 50 nM RAB11A siRNA for 48 h. **a, b** Interference efficiencies of RAB11A siRNA were determined by qRT-PCR and Western blotting, respectively. Subsequently, LNCap cells were transfected with 2 μg/mL pcDNA-NORAD alone or together with 50 nM RAB11A siRNA. **c** After 48 h transfection, CCK-8 was used to detect the cell proliferation. **d, e** Cell apoptosis and invasion was determined by Flow cytometry and Transwell assay in LNCap cells after 48 h transfection with NORAD alone or together with RAB11A siRNA. **f, g** Western blotting was used to determine the expression levels of related proteins of EMT (N-cadherin, E-cadherin, Vimentin and Snail) and WNT pathway (β-catenin, Cyclin D and c-Myc) in LNCap cells after 48 h transfection. The data were presented as the mean ± standard error of mean (SEM), n=3. Student’s t test was used for the comparison between 2 groups in this study. * *P* < 0.05


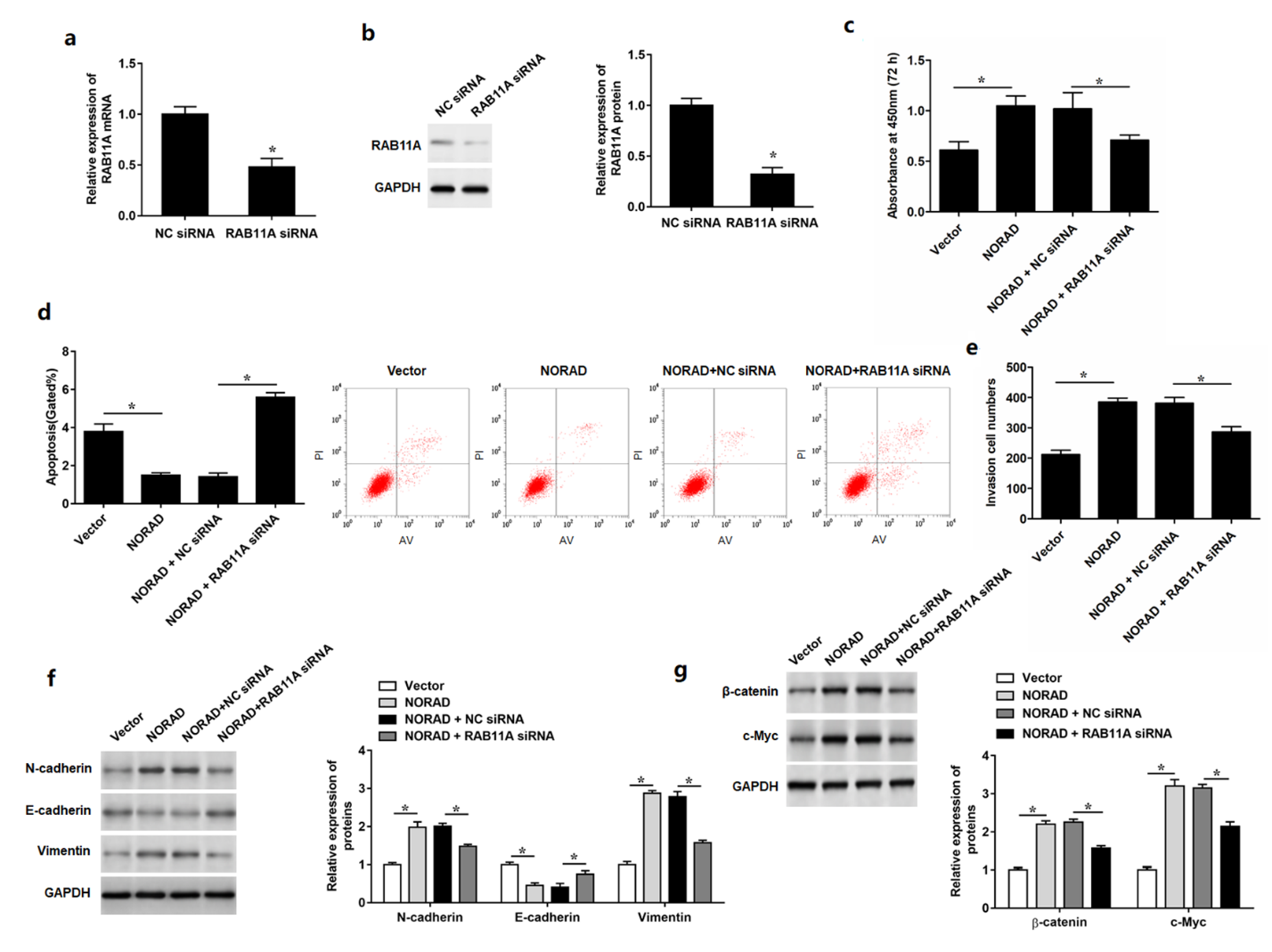

Supplement: Supplementary file 6 — Additional file 6: Figure S5. NORAD promotes cell proliferation, invasion and EMT, suppresses cell apoptosis via miR-30a-5p/RAB11A/WNT/β-catenin pathway in LNCap cells. [file 12935_2020_1665_MOESM6_ESM.docx]
